# The Whistle‐Smile Reflex in Parkinson's Disease: A Cross‐Sectional Study

**DOI:** 10.1002/mdc3.14175

**Published:** 2024-07-31

**Authors:** Giulia Paparella, Luca Marsili, Kevin R. Duque, Lucrezia Balestrucci, Adriana Martini, Antonio Cannavacciuolo, Davide Costa, Daniele Birreci, Martina De Riggi, Luca Angelini, Alberto J. Espay, Matteo Bologna

**Affiliations:** ^1^ Department of Human Neurosciences Sapienza University of Rome Rome Italy; ^2^ IRCCS Neuromed Pozzilli Italy; ^3^ James J. and Joan A. Gardner Family Center for Parkinson's Disease and Movement Disorders, Department of Neurology University of Cincinnati Cincinnati Ohio USA

**Keywords:** Parkinson's disease, facial bradykinesia, hypomimia, facial expressiveness, whistle‐smile reflex

Parkinson's disease (PD) is a neurodegenerative disorder characterized by motor and nonmotor symptoms, including facial abnormalities such as hypomimia, which significantly impacts social interactions and life quality in patients.^1^ Hypomimia, also known as ‘facial bradykinesia',^1^ denotes a diminished facial expressiveness, encompassing reduced spontaneous blinking, decreased eyelid/eyebrow movements, and a reduction in facial movements involving the lower part, for example, grimacing and smiling.^1^, ^2^ In this context, the “whistle‐smile” reflex (WSR), or Hanes sign, was first described in 1943 by Dr. Frederic Hanes, who observed that when healthy individuals are prompted to whistle during a medical examination, they typically comply and subsequently smile, reacting to the request's incongruity.^3^ However, when PD patients receive the same instruction, they execute the whistle but often fail to produce an immediate smile. Despite its potential diagnostic value, the WSR has seen limited clinical use, and only 2 reports, including Hanes's original work, explored this sign in PD.^3^, ^4^

In the present study, we examined the WSR in a broad sample of PD patients compared to age‐ and gender‐matched healthy controls (HCs) to evaluate its sensitivity and specificity as a diagnostic marker. We enrolled 34 PD patients (assessed with their regular dopaminergic therapy) and 34 HCs (Table S1). The study was approved by local ethics committees, and informed consent was obtained from participants. Clinical evaluations included standardized scales. Video recordings followed standardized protocols (Supplementary Materials: Data S1). Briefly, participants' faces were recorded in a relaxed state for 30 seconds while looking at the camera (spontaneous facial expression videos). Participants were then asked to perform a whistle, and the recording continued for the next 15 s (WSR videos). Offline video editing was performed to ensure a blinded assessment (Supplementary Materials: Data S1). Videos were randomized and presented to 4 experienced movement disorders evaluators, unaware of the participants' diagnoses. Evaluators rated the severity of hypomimia per the Movement Disorders Society‐sponsored version of the Unified Parkinson's Disease Rating Scale (MDS‐UPDRS), item 3.2, as well as the WSR, indicating whether they observed an immediate smile after whistling. Inter‐rater agreement was computed using Fleiss’ K coefficient. Further data analyses are included in Supplementary Materials: Data S1. The overall agreement among raters in the assessment of the spontaneous facial expression videos was moderate (Fleiss’ K value: 0.45). We found a higher score in item 3.2 of the MDS‐UPDRS scale Part III in PD patients compared to HCs (Supplementary Figure S1). The overall agreement among raters in the WSR video evaluation was substantial (Fleiss’ K value: 0.73). We found that 76.47% of PD patients did not exhibit a smile after whistling, compared to 35.29% of HCs (Video 1 and Supplementary Figure S2). The WSR showed a sensitivity of 76% and a specificity of 65% as a PD diagnostic marker. Logistic regression analysis revealed a correlation between the absence of WSR and the severity of hypomimia but not with cognitive or emotional impairment (Supplementary Materials: Data S1). The present study demonstrates the absence of the WSR in most of the PD patients tested employing a validated experimental approach and suggested it as a feature of an aberrant facial expressiveness automaticity. Taking into account some limitations inherent to our experimental approach (see Supplementary Materials: Data S1), the WSR could be an additional clinical sign to use in patient assessment, possibly in the early or prodromal disease phases.^5^ Further research is needed to validate the results of the present study.

**Video 1:** The video shows examples of the whistle‐smile reflex (WSR) in 2 distinct groups: healthy controls (HCs) and patients diagnosed with Parkinson's disease (PD). In segment 1, a HC exhibits a clear smile after whistling, as confirmed by the high agreement among the 4 raters who conducted the blinded WSR video assessment. This response was observed in 22 of 34 HCs (64.71%) included in the study. Segment 2: a HC does not exhibit a smile after whistling, as agreed upon by the 4 raters. This scenario was observed in only 12 of 34 HCs (35.29%). Segment 3 shows a PD patient who does not smile after whistling, confirmed by high agreement among raters. This response was noted in 26 of 34 PD patients (76.47%). Finally, segment 4 shows a PD patient who clearly smiles after the whistle. This response was noted in only 8 of 34 PD patients (23.53%). It is important to note that while the original recordings were included in the present video, the raters' evaluation was conducted on edited videos. These edited videos focused only on the mouth area, excluding the upper face and shoulders of the participant. This measure ensured that evaluators were not influenced by potential facial hypomimia or postural alterations indicative of the diagnosis.

## Author Roles

(1) Research project: A. Conception, B. Organization, C. Execution; (2) Statistical analysis: A. Design, B. Execution, C. Review and critique; (3) Manuscript: A. Writing of the first draft, B. Review and critique.

G.P.: 1B, 1C, 2A, 2B, 3A

L.M.: 1B, 1C, 3B

K.R.D.: 1C, 3B

L.B.: 1B, 1C

A.M.: 1C

A.C.: 1B, 1C

D.C.: 1C

D.B.: 1C

M.R.: 1C

L.A.: 2A, 2B

A.J.E.: 3B

M.B.: 1A, 1B, 2C, 3B

## Disclosures

**Ethical Compliance Statement:** This study was approved by the local ethics committee and conducted in accordance with the principles outlined in the Helsinki Declaration. All participants provided written informed consent to participate in the study. All authors have read the journal's position on issues involved in ethical publication and affirm that this work is consistent with those guidelines.

**Funding Sources and Conflicts of Interest:** This work was supported by the Italian Ministry of Health (Current Research 2024). The authors declare that there are no conflicts of interest relevant to this work.

**Financial Disclosures for the Previous 12 Months:** The authors declare that there are no additional disclosures to report. Dr. Luca Marsili has received honoraria from the International Association of Parkinsonism and Related Disorders (IAPRD) Society for social media and web support, and personal compensation as a consultant/scientific advisory board member from Acadia. Dr. Marsili has received a grant (collaborative research agreement) from the International Parkinson and Movement Disorders Society for the MDS‐UTRS Validation Program (Role: PI), Non‐Profit. Dr. Alberto J. Espay has received grant support from the NIH and the Michael J. Fox Foundation; personal compensation as a consultant/scientific advisory board member from Neuroderm, Amneal, Acadia, Avion Pharmaceuticals, Acorda, Kyowa Kirin, Supernus (formerly USWorldMeds), NeuroDiagnostics, Inc (SYNAPS Dx), and Herantis Pharma; and publishing royalties from Lippincott Williams & Wilkins, Cambridge University Press, and Springer. He cofounded REGAIN Therapeutics and is coinventor of the patent “Compositions and Methods for Treatment and/or Prophylaxis of Proteinopathies.”

## Supporting information


**Data S1.** Supplementary methods: details of participants, clinical and video evaluation, and statistical analysis are provided in the supplementary materials, along with the main study results and limitations.
**Figure S1.** Movement Disorders Society‐sponsored revision of the Unified Parkinson's Disease Rating Scale (MDS‐UPDRS), Part III, item 3.2 scores in the 2 groups of participants as blinded evaluation by 4 raters.
**Figure S2.** Number of participants (percentages in brackets) belonging to the Parkinson's disease (PD) and healthy controls (HCs) groups who smiled (white) or did not smile (black) after the whistle according to at least 3 of 4 raters.
**Table S1.** Clinical‐demographic data in patients with Parkinson's disease (PD) and healthy controls (HCs).

## Data Availability

The data that support the findings of this study are available on request from the corresponding author. The data are not publicly available due to privacy or ethical restrictions.
