# Supplementary material for: The Whistle‐Smile Reflex in Parkinson's Disease: A Cross‐Sectional Study
Source: Mov Disord Clin Pract. 2024 Jul 31;11(10):1314–6. doi: 10.1002/mdc3.14175 (PMC11489615; doi:10.1002/mdc3.14175)
Supplement: Supplementary file 1 — Data S1. Supplementary methods: details of participants, clinical and video evaluation, and statistical analysis are provided in the supplementary materials, along with the main study results and limitations. Figure S1. Movement Disorders Society‐sponsored revision of the Unified Parkinson's Disease Rating Scale (MDS‐UPDRS), Part III, item 3.2 scores in the 2 groups of participants as blinded evaluation by 4 raters. Figure S2. Number of participants (percentages in brackets) belonging to the Parkinson's disease (PD) and healthy controls (HCs) groups who smiled (white) or did not smile (black) after the whistle according to at least 3 of 4 raters. Table S1. Clinical‐demographic data in patients with Parkinson's disease (PD) and healthy controls (HCs). [file MDC3-11-1314-s001.docx]

**SUPPLEMENTARY MATERIALS - THE WHISTLE-SMILE REFLEX IN PARKINSON'S DISEASE: A CROSS-SECTIONAL STUDY**

**METHODS AND MATERIALS**

**Participants**

We consecutively recruited 34 patients diagnosed with PD according to the Movement Disorders Society (MDS) criteria, and 34 age- and gender-matched HCs, at the Movement Disorders Outpatient Clinics, Department of Human Neuroscience, Sapienza University of Rome, and at the Gardner Family Center for Parkinson's Disease and Movement Disorders, Department of Neurology, University of Cincinnati, Cincinnati, OH, USA, from January to December 2023. All participants were right-handed, as evaluated by the Handedness Questionnaire. Exclusion criteria involved atypical or secondary parkinsonism, severe cognitive impairment, mood depression or other psychiatric disorders explored by a clinical interview, and the use of anxiolytics or antidepressants. None of the HCs had a history of neurological or psychiatric disorders, nor were they taking any medications acting on the nervous system. Participants underwent assessment during a single experimental session. PD patients were evaluated while on their regular dopaminergic therapy. Informed consent was obtained from all participants for their involvement in experimental procedures, which were approved by the local Ethics Committee and conducted in accordance with the principles outlined in the Helsinki Declaration.

**Clinical Evaluation**

Information regarding demographic characteristics, medical history, and disease course was collected through direct interviews with study participants. All participants underwent a comprehensive neurological examination. We assessed patients using the MDS-sponsored version of the Unified Parkinson's Disease Rating Scale parts II and III (MDS-UPDRS-III), the Non-Motor Symptoms Scale for Parkinson’s Disease (NMSS), as well as the Parkinson’s Disease Quality of Life Questionnaire (PDQ-8). Additionally, all participants were assessed using the following clinical scales to evaluate cognitive and psychiatric aspects: Montreal Cognitive Assessment (MoCA), Beck Anxiety Inventory (BAI), Beck Depression Inventory-I (BDI-I).

**Video Recording and Evaluation**

Video recordings adhered to standardized protocols. Participants were seated without head support, feet resting on the floor, and hands at rest. A neutral background was positioned behind the subject. Participants' faces were recorded in a relaxed state for 30 seconds while looking at the camera (spontaneous facial expression videos). Using a standard phrase, the study participants were then asked to perform a brief whistle, and the recording continued for the next 15 seconds. Recording encompassed the upper body, including the head, hair, and shoulders, with a high-resolution camera situated approximately 60 cm from the subject. Offline editing of the videos included sound removal, and, for the WSR videos, cropping the upper part of the face and shoulders of the participant, leaving only the mouth area visible for blinded assessment. This measure ensured that subsequent evaluators were not influenced by potential facial hypomimia or postural alterations indicative of PD diagnosis. Subsequently, the videos were randomized into two blocks (spontaneous facial expression and WSR) and presented to four experienced evaluators in movement disorders, unaware of the participants' diagnoses. During the evaluation of the spontaneous facial expression videos, each evaluator rated the severity of hypomimia on a scale from 0 to 4, as per the instructions of item 3.2 of part III of the MDS-UPDRS scale. Specifically, a score of 0 indicated normal facial expression, 1 denoted minimal fixed facial expression with reduced spontaneous blinking, 2 represented reduced blinking and fixed facial expressions in the lower part of the face, including reduced spontaneous smiling but closed lips, 3 indicated fixed facial expressions with occasional open lips when the mouth was still, and 4 reflected fixed facial expressions with open lips most of the time when the mouth was inactive. For each subject, we proceeded by considering the median of the ratings provided by the four raters. During the evaluation of the WSR videos, raters indicated with a binary code (0: yes; 1: no) whether they observed an immediate smile after whistling. After each choice, the raters were also asked to grade their confidence level in the accuracy of their response, from ‘0’—‘no confidence at all’, to ‘10’—‘maximum confidence’. To avoid any bias associated with the viewing of the entire face, raters were asked first to assess videos depicting only the lower half of the face to determine whether participants were smiling. Once more, the median of the ratings from the four raters was taken into account for each participant.

**Statistical Analysis**

Clinical and demographic data of PD patients and HCs were compared using non-parametric tests, including Mann-Whitney test and Fisher's test. We calculated the agreement among evaluators in video assessments using Fleiss’ K coefficient. We compared the data of video assessments between PD patients and HCs using non-parametric tests. Additionally, we assessed the sensitivity and specificity, as well as positive and negative predictive values, of the WSR impairment as a diagnostic marker for PD. Specifically, we considered the WSR preserved in participants when at least three out of four raters, during blind video evaluations, noted the occurrence of a smile following the whistle. Conversely, we deemed the WSR impaired or absent when at least three out of four evaluators reported the absence of a smile following the whistle. Finally, we test the possible relationship between WSR (presence/absence) and clinical data using regression analyses. We conducted a multiple logistic regression in the whole sample of participants using the presence or absence of the WSR as dependent variable, and the MDS-UPDRS part III item 3.2 scores obtained at the spontaneous facial expression videos evaluation, age, MoCA, BDI and BAI scores as independent variables. Another multiple logistic regression was conducted considering only PD data, with the same dependent variable, and disease duration, age at onset, MDS-UPDRS Part III and II, PDQ-8 and NMSS scores as further independent variables. Variance Inflation Factor (VIF) and tolerance were used to assess multicollinearity. Unless otherwise stated, results are indicated as mean values ± 1 standard deviation (SD). Results were considered significant for p < 0.05.

**RESULTS**

The groups were similar in age (mean age ± SD: PD: 70.23 ± 10.1 years; HCs: 69.73±8.35 years) and sex (PD: 10 female - F; HCs: 15 F; Supplementary Table 1). In PD patients, the mean disease duration was 7.11 ± 6.29 years, with a mean age of disease onset of 63.91 ± 11.28 years. Other clinical data are reported in Supplementary Table 1.

**Clinical Evaluation**

The total scores of the Part III of the MDS-UPDRS in PD patients were 26.1 ± 15.5 (Supplementary Table 1). The MDS-UPDRS Part II scores were 4.4 ± 4.6 in PD (Supplementary Table 1). The were no differences in MoCA, BAI and BDI scores between the two groups (Supplementary Table 1).

**Video Evaluation**

*Spontaneous facial expression*

There was a higher score in item 3.2 of the MDS-UPDRS scale Part III in PD patients compared to HCs [median PD (IQR): 2 (0), HCs: 0.25 (0.5), p<0.001] (Supplementary Figure 1). The overall agreement among evaluators in the assessment of the spontaneous facial expression videos was moderate, as indicated by the Fleiss’ K value: 0.45. Specifically, the Fleiss K was 0.19 for the PD group (slight agreement) and 0.35 (fair agreement) for HCs.

*WSR*

Twenty-six out of 34 PD patients (76.47%) did not exhibit a smile after whistling according to at least three out of four evaluators. In contrast, only 12 out of 34 HCs (35.29%) failed to smile after whistling according to the evaluators (p=0.001, Chi-square test) (Supplementary Figure 2). Eight PD patients out of 34 (23.53%) and 22 out of 34 HCs (64.71%) consistently smiled after whistling, as per at least three out of four evaluators (Supplementary Figure 2). Video 1 provides some examples of the WSR in PD patients and HCs.

The overall agreement among raters in the evaluation of ‘WSR’ videos was substantial, as indicated by the Fleiss’ K value: 0.73. Specifically, the Fleiss K was 0.76 for the PD group and 0.65 for HCs. There were no significant differences in raters' responses confidence level in the evaluation of the ‘WSR’ videos between PD and HCs groups [median confidence level (interquartile range - IQR) in PD: 7.5 (1.5), HCs: 7 (1.5), p=0.91]. The sensitivity, specificity, positive predictive value, and negative predictive value of the absence of the WSR in differentiating PD patients from HCs were found to be 76%, 65%, 68%, and 73%, respectively.

**Logistic regression analysis**

In the multiple logistic regression performed on the whole participants sample, the model was statistically significant, χ2(1)=18.05, p=0.003, with 19.3% of variance explained, and 70.6% of subjects correctly classified in smiling after whistling or not. Among the factors examined, only the MDS-UPDRS part III item 3.2 as blinded evaluated exhibited statistical significance (p=0.002). Specifically, increased scores were associated with a reduced probability of the presence of the WSR (odds ratio=0.395, 95% CI 0.218-0.716). This implies that individuals who received higher scores in the blinded assessment were less likely to smile after whistling. Other demographic and clinical factors were not correlated with the presence of the reflex in participants (age: odds ratio=0.927, 95% CI 0.858-1.002, MoCA: odds ratio=0.877, 95% CI 0.731-1.051, BDI: odds ratio=1.049, 95% CI 0.964-1.142, BAI: odds ratio=0.990, 95% CI 0.916-1.070).

The multiple logistic regression performed considering only PD data did not show any other significant relations between the presence of the WSR and disease duration, age at onset, MDS-UPDRS Parts III and II scores, PDQ-8 nor NMSS scores (χ2(1)=5.94, p=0.43; R^2^_McF_=0.16).

**STUDY LIMITATIONS**

The sample of enrolled participants is relatively small, although it currently represents the largest sample on which the WSR has been investigated thus far. Furthermore, we concentrated on patients in the moderate phase of the disease, emphasizing the need for studies across different disease stages, including individuals with newly diagnosed PD or drug naïve, and longitudinal studies to validate our findings. While MoCA served as our primary measure of cognition, it's important to note that it may not comprehensively assess executive functions. Moreover, despite excluding subjects with depression or other psychiatric disorders, which could impact the interpretation of our findings, we didn't assess apathy. These aspects warrant consideration in our analysis. While the overall agreement among evaluators in assessing the spontaneous facial expression videos was moderate, as indicated by the Fleiss’ K value of 0.45, it is noteworthy that the agreement was only slight when evaluating spontaneous facial expression videos within the PD group. This observation should be carefully considered, especially when interpreting the correlation between hypomimia and WSR absence. Finally, in our study, we utilized the term 'reflex' within the context of the WSR, aligning with the established nomenclature. However, we acknowledge that 'reflex' typically denotes a motor reaction occurring involuntarily, triggered by a sensory stimulus, without conscious thought. In the case of the WSR, the underlying mechanism revolves around the incongruity of requesting someone to whistle during an examination, which elicits a smile. Therefore, in hindsight, employing the term 'response' rather than 'reflex' would have been more accurate.

**SUPPLEMENTARY FIGURES**

**Supplementary Figure 1**

**

**

**Supplementary Figure 2**





**Supplementary Table 1**

|  | **PD (n= 34)** | **HCs (n= 34)** | **P Value** |
| --- | --- | --- | --- |
| Gender female | 10 (29.4%) | 15 (44.1%) | 0.15 |
| Age | 70.23±10.1 | 69.73±8.35 | 0.9 |
| Disease duration | 7.11±6.29 | - | - |
| Age at disease onset | 63.91±11.28 | - |  |
| MoCA | 25.94±3.2 | 25.06±3.82 | 0.27 |
| BAI | 6±9.96 | 3.26±7.14 | 0.06 |
| BDI | 5.38±6.02 | 4.32±8.28 | 0.06 |
| MDS-UPDRS Part III (ON condition) | 26.11 ± 15.54 | - | - |
| MDS-UPDRS Part II (ON condition) | 4.44±4.59 | - | - |
| PDQ-8 | 5.7±8.59 | - | - |
| NMSS | 32.94±32.65 | - | - |
| LEDD | 528.9±273.93 | - | - |
